# Noise Resilient Outdoor Traffic Light Visible Light Communications System Based on Logarithmic Transimpedance Circuit: Experimental Demonstration of a 50 m Reliable Link in Direct Sun Exposure

**DOI:** 10.3390/s20030909

**Published:** 2020-02-08

**Authors:** Sebastian Andrei Avătămăniței, Alin-Mihai Căilean, Adrian Done, Mihai Dimian, Marius Prelipceanu

**Affiliations:** 1 Integrated Center for research, development and innovation in Advanced Materials, Nanotechnologies, and Distributed Systems for fabrication and control, Stefan cel Mare University of Suceava, 720229 Suceava, Romania; sebastian.avatamanitei@usm.ro (S.A.A.); adone@eed.usv.ro (A.D.); dimian@usm.ro (M.D.); mprelipceanu@eed.usv.ro (M.P.); 2 Department of Computers, Electronics and Automation, Stefan cel Mare University of Suceava, 720229 Suceava, Romania

**Keywords:** noise, logarithmic transimpedance circuit, inter-vehicle communications, visible light communications

## Abstract

The usage of Visible Light Communications (VLC) technology in automotive applications is very promising. Nevertheless, in outdoor conditions, the performances of existing VLC systems are strongly affected by the sun or other sources of light. In such situations, the strong parasitic light can saturate the photosensitive element and block data communication. To address the issue, this article analyzes the usage of an adaptive logarithmic transimpedance circuit as an alternative to the classical linear transimpedance circuit. The simulation and experimental evaluation demonstrate benefits of the proposed technique, as it significantly expands the communication distance and optical noise functionality range of the VLC systems and reduces the possibility of photoelement saturation. As a result, this approach might enable outdoor VLC sensors to work in strong sun conditions, the experimental results confirming its validity not only in the laboratory but also in outdoor conditions. A reliable 50 m communication distance is reported for outdoor sunny conditions using a standard power traffic light VLC emitter and a PIN photodiode VLC sensor.

## 1. Introduction

Visible Light Communications (VLC) represent a new wireless communication technology which transforms a basic LED lighting device into an intelligent dual purpose tool used for lighting and communications simultaneously [1,2,3,4]. VLC is based on the solid-state lighting devices’ fast switching ability, which enables them to be used in communication purposes as well. So, in VLC, the communication feature is achieved by modulating the data onto the instantaneous power of the optical carrier at rates that are imperceptible for the human eye [5]. The data communication is thus achieved without perturbing in any way the primary function of the lighting device and without using any extra power to generate the carrier wave. In these conditions, the VLC technology is favored by the wide distribution of light sources. Due to the incontestable competitive advantages, existing research forecast that LED lighting sources are on the way to totally replacing classical lighting devices [6,7,8]. Consequently, from a hardware point of view, the VLC technology can be viewed as half implemented worldwide, since a data processing unit and a digital power switch are all that an LED illumination source needs in order to be transformed into a data broadcasting device for indoor and outdoor applications.

Regarding the usage of VLC technology in automotive applications, this domain is favored by the integration of the LED light sources as part of the traffic infrastructure (i.e., traffic lights, street lighting system, traffic signs, traffic panels) and also within vehicle lighting systems [2,9]. This fact is very important because the performance and efficiency of an intelligent transportation system based on wireless communications are given by the technology’s geographical distribution and penetration rate [10]. From this point of view, VLC is able to provide a low-cost solution for fast deployment and wide area distribution. Thus, the VLC technology could be used to enable both traffic Infrastructure-to-Vehicle (I2V) and Vehicle-to-Vehicle (V2V) communications [11,12,13,14,15]. Nevertheless, supporting vehicular communications is rather challenging due to the strict requirements (i.e., latencies below 20 ms and high packet delivery ratio) [2,5]. Major challenges are related to the noisy outdoor VLC channel [15,16,17] and the characteristics of vehicular communications [2]. In the outdoor scenario, the light incident on the VLC sensor contains not only the data optical signal, but also other strong sources of natural and artificial light [17]. Thus, in the worst-case scenario, when the sun is directly facing the VLC sensor, the irradiance of the incident sunlight can reach a very high level which strongly affects the packet delivery ratio. Although influence of the parasitic light can be reduced using different solutions [2,18,19,20,21,22], a proportional shot noise component is still introduced in the system. Consequently, in daytime conditions, the background light represents the most important perturbing element for VLC usage in vehicular applications, especially when medium range communications are envisioned. Vehicular VLC applications require communication ranges up to few tens of meters or even more, [2], whereas the irradiance of the received data signal decrease proportionally to the square of the distance. In such circumstances, the irradiance of the received light can decrease at nW/cm^2^ levels. Furthermore, unlike in other technologies, the emitted power cannot be increased as much as necessary because it is strictly dependent on the VLC emitter’s primary purpose [5]. Thus, a data broadcasting LED traffic light or a vehicle LED tail lamp should have the same optical power as they were initially designed and limited by automotive lighting standards, even though they had been enhanced with data transmitting capabilities. In this context, automotive VLC systems should be able to provide highly resilient communications under very low Signal to Noise Ratio (SNR) conditions. Therefore, enhancing the robustness to noise and increasing the communication distance represent two of the main challenges for automotive VLC systems developers [2].

To address these issues, an adaptive logarithmic transimpedance circuit is proposed instead of the classical linear approach. As far as we know, this is the first intensive experimental investigation of such a concept in outdoor VLC applications. The simulation results and the experimental tests have confirmed that in comparison with the linear transimpedance circuit, the adaptive logarithmic approach provides the VLC receiver with an extended dynamic range and improved robustness to photoelement saturation, in accordance with the requirements of automotive applications [2]. Thus, the integration of the logarithmic transimpedance circuit enables usage of VLC sensors in automotive applications not only in favorable conditions but also problematic circumstances. Usage of the logarithmic transimpedance circuit for VLC sensors has been partially addressed by our group in [23]. However, unlike in [23], this article provides the results of extensive simulation and experimental testing and evaluation that confirm the performances of the prototype, more insights on the concept, and details on the benefits and applications.

The rest of this article is structured as follows: Section 2 addresses the issues associated to the usage of the VLC technology in vehicular applications and presents some of the existing solutions to mitigate them. Section 3 introduces the concept of adaptive logarithmic transimpedance and presents the advantages associated with its usage in automotive VLC applications. Section 4 summarizes the results of the simulation and of the experimental evaluation of a logarithmic transimpedance circuit providing a comparison to the linear approach. Section 5 provides a discussion on the observations that can be drawn from the experimental evaluation and discusses the future perspectives in this direction, whereas Section 6 provides the final conclusions of this article, followed by a bibliography.

## 2. Debate on the Usage of the Visible Light Communication Technology in Automotive Applications: Issues and State of the Art Solutions

### 2.1. Optical Interferences Associated Issues and Solutions

Whether we like it or not, autonomous vehicles will become a reality in the near future as part of the Intelligent Transportation System and Smart City concepts [24]. This solution is based on the fact that such vehicles have the potential to provide enhanced safety and improved traffic efficiency. In this new vehicular paradigm, wireless communications will play one of the leading roles, as they have the potential to provide the vehicle with much more information than any other on-vehicle sensor. Moreover, wireless communication technologies will enable a vehicle to have information concerning an event that occurred few kilometers away from its current location. Nevertheless, there are several wireless access technologies that compete in order to have their market share in this new emerging industry. Currently, IEEE 802.11p and short-range Cellular-V2X are some of the most advanced technologies in this domain, whereas other technologies, such as VLC, millimeter Waves, or 5G technologies are eager to prove their potential in this field [25].

The usage of VLC technology in outdoor applications implies significantly different conditions compared to its usage in indoor applications. Thus, in indoor applications, the VLC emitter is assumed to be the primary source of light, and so high SNRs that can go up to 20–50 dB are expected [26]. In these circumstances, it is assumed that the indoor lighting system is used in VLC, and so perturbations from artificial lighting are not implicated. So, the high SNR indoor conditions enable the usage of the VLC technology in high data rate applications, coined today as Li-Fi technology [26,27], high precision indoor location [28,29,30], Internet of Things (IoT) services [31], location based informational systems [32], smartphone applications [33], and future 5G/6G technologies [26,34]. On the other hand, outdoor applications are affected by numerous sources of artificial light. Incandescent lighting sources and fluorescent lighting sources introduce a strong 100 Hz component with harmonics that can go up to 2 kHz, respectively 20 kHz. Moreover, fluorescent lighting sources with electronic ballast introduce harmonics that can go up to few MHz [2]. In addition to the multitude of artificial lighting sources, the sun introduces a strong light component that can go up to 100.000 lux. Based on the existing outdoor VLC literature [2,16,35,36] and on the arguments presented in Section 1, strong sunlight is considered the most important source of noise in outdoor VLC applications. The effects of the sunlight are influenced by location and time, with the effects on different spectral bands being modified from sunrise to sunset [16].

An attempt to develop an automotive VLC system designed to work in direct sun exposure is presented in [37]. In this case, the sensibility of the transimpedance circuit was established in order to prevent saturation of the photosensitive element in direct sun exposure (i.e., 100.000 lux). This approach enabled the communication in strong sunlight conditions, but reduced the reliable communication link to only 10–12 m. A 50 m communication distance has been reported in [20]. However, in this case, the experimental evaluation is performed in outdoor conditions without the sunlight being directly incident on the VLC receiver. In [38], the effect of the optical noise is reduced with the help of a spread spectrum modulation. Nevertheless, the spread spectrum techniques are helpful only if the photosensitive element is not saturated, pointing out that solutions to prevent the saturation of the automotive VLC receivers are still required. Probably the most impressive results concerning the VLC usage in strong lighting conditions are found in [16,36]. According to the findings of these papers [16,36], high data rate communications that can go up to 1 Gb/s are possible even in direct sunlight exposure. Although the authors are mainly focused on the indoor use case, the resilience to noise and the data rate of this VLC system is higher than the one of any existing outdoor VLC system. Nevertheless, the simulation results further show that as the distance increases from 1 to 3 m, the data rates are reduced to half of the initial values, pointing out that the sunlight effect is highly disruptive. To improve these results, the authors propose the usage of a narrow band spectrum for the link, associated to the usage of narrow band optical filters. Even if this solution improves the link performances, its usage in automotive applications seems rather inappropriate. Unlike in indoor applications which use the blue component of the white light for high speed communications [26], in automotive applications this solution would require multiple VLC sensors, each with its narrow band optical filter (i.e., red, green, and orange band for the reception of signals coming from traffic lights, blue band for the reception of signals coming from a vehicle headlamp or from the street lighting system). Thus, although it has the potential to improve the SNR and the associated system performance, the complexity and the cost of the system are increased by at least four times. Moreover, when the transition from simulations to experimental evaluation is made, the communication distance in strong lighting conditions (i.e., 50,350 lux) is significantly reduced to 0.14 m, pointing again to the importance of real-life experimental testing in concept evaluation and validation in this area.

To reduce the effect of ambient noise and to improve the SNR, the authors of [39] propose the usage of an average voltage detector along with a voltage subtractor that eliminates part of the generated noise. The SNR improvement is confirmed experimentally in an indoor scenario. Although the solution is effective in terms of SNR and Bit Error Rate (BER) improvement, again, its usage in an outdoor scenario can be effective only if the transimpedance circuit is not saturated by the sun. Another interesting and promising solution to improve the performances of VLC systems in strong sunlight conditions is proposed in [40] and is based on the concept of optical interference suppression based on LCD filtering. Unlike the previous solutions, this one has the potential to contribute to the mitigation of the photosensitive element saturation possibility but this solution proposes a new concept of active filtering rather than a new VLC receiver.

### 2.2. Meteorological Conditions Associated Issues and Solutions

In addition to the optical interference, automotive VLC applications are also affected by the mobility and unpredictability of the outdoor VLC channel [2]. Due to its intrinsic nature, vehicular applications involve frequent and unexpected direction changes, highly variable inter-vehicle distances, highly variable velocities, and so on. Thus, as the VLC technology requires a mandatory Line of Sight (LoS), connectivity problems might arise. Additionally, the variable emitter receiver distance significantly influences the power of the received optical signals. To compensate the input power variance, automotive VLC receivers usually integrate an Automatic Gain Control (AGC) stage [2]. In addition, the unpredictability of the vehicular VLC channel is further amplified by the unpredictability of the multitude of meteorological conditions and phenomena that can influence the passage of the optical data carrier. Thus, the water particles from rain or fog can influence the light passage through a combination of reflection, refraction, absorption, and scattering [2,11,15,41,42], whereas the snow or heavy dust block part of the optical channel [2,42,43]. All these phenomena eventually lead to a lower optical power detected by the VLC receiver [2,11,15,41,42,43], which in turn affect the SNR, the communication distance, and the BER. Based on the simulation results performed in [15], the authors consider that the heavy fog reduces the maximum V2V communication distance by more than 60%. The high impact of fog is also determined experimentally in [41] for a 5.5 m V2V VLC link. In this case, the results also show that the attenuation of the light is higher as the modulation frequency is increasing. This work also determines the fact that the attenuation introduced by the heavy fog is stronger than the one introduced by the rain or the thermal turbulences. To address the problems associated with heavy fog, the authors of [11] considered the usage of a Fresnel lens and multiple photodiodes. The experimental results performed at 1 m in laboratory conditions demonstrated that the proposed approach enables a VLC system to have a reliable connection in a fog-impaired optical channel and a relatively high SNR. The authors of [42] performed a simulation analysis concerning the effect of rain and snow on I2V VLC, showing that the attenuation introduced by snow is higher than the one due to rain for the same precipitation rate. The results also showed that the communication distance of an I2V VLC link can be decreased by 20%–80% due to snow and by 8%–30% due to rain.

In the upper-mentioned context, one can see that the adverse meteorological conditions affect VLC mainly by introducing a signal attenuation which depends on the meteorological phenomena and its intensity. Nevertheless, from a VLC developer point of view, one cannot contribute to the reduction of the attenuation factor introduced by any of these phenomena. A possible solution to increase the optical power level is to use optical lens and multiple photosensitive elements as suggested in [11]. It is expected that future work concerning the effect of meteorological conditions on automotive VLC would be focused on developing highly accurate SNR prediction models in order to have the most exact data concerning the power of the received optical signal. Based on such information, future automotive VLC systems could use artificial intelligence and neural networks [44] to develop high performance environment-adaptive VLC systems [22].

Table 1 provides the summary of the issues associated to the usage of VLC in vehicular applications and of some of the existing solutions to address them. One can see that from a hardware approach, there is much more to be done in terms of increasing the VLC systems robustness to light interferences rather than on mitigating the signal attenuation due to meteorological phenomena. Moreover, it is obvious that by developing a VLC receiver that is highly resilient to light interferences, the SNR is enhanced and so, the overall system performances are improved, including here the performances achieved in adverse meteorological conditions. Based on this analysis, this article continues with the aspects associated to the development and the evaluation of a new VLC receiver concept based on a logarithmic transimpedance solution.

### 2.3. Long-Range Vehicular Communications State-of-the-Art

Concerning the communication distances, one can see that the above mentioned works present results mainly determined based on simulations. For the cases when experimental results are reported, the testing setups are generally laboratory setups with demonstrational prototypes tested at relatively short distances [11,16,41]. In other cases, these works present outdoor experimental results in favorable conditions [20,38], or they present rather short communication distances [37].

In recent years, interest in the usage of VLC technology in vehicular applications has increased significantly. This has led to the development of numerous vehicular VLC systems. Nevertheless, a problem with some of the existing VLC prototypes comes from the fact that in most cases researchers aim to achieve top performances focusing on one specific issue while almost completely ignoring other important factors. For example, in order to increase the data rate of vehicular VLC systems to unprecedented values, the communication distance and the robustness to noise are left aside. Thus, the V2V VLC systems presented in [45,46] are able to achieve data rates up to 427 Mb/s, but are only able to achieve a 1.2–2.5 communication distance while using a narrow beam optical signal.

On the other hand, the vehicular VLC systems presented in [47,48,49,50,51,52] are developed while considering the communication range as the main priority. Again, one can see that even if the communication distance is significantly increased, in most cases the experimental testing procedure is totally different compared to the real life use case. So, many of these systems are evaluated in rather friendly conditions, compared to a worst case scenario imposed for vehicular applications. So, referring to communication distances, the VLC system presented in [47] is able to achieve a range of 70–80 m at a bit rate of 30 b/s in the absence of direct incident light by using a low cost camera based VLC receiver. Also based on a camera VLC receiver, the authors of [48] report a 100 m communication range using off-line data processing. The authors of [49] aim to determine the maximum communication distance that could be achieved using a camera-based VLC receiver approach, in non-LoS conditions. Based on an analytical process and on simulations, the authors consider that such systems could achieve a 2000 m range. Nevertheless, their experimental evaluation showed a range of 300 m, significantly lower than the range indicated by the simulations. Although this work is not referring to vehicular VLC, it points out again that in general, experimental evaluations are lower in performance than the results provided by simulations.

The system presented in [50] is able to provide an effective I2V transmission for a 66 m communication distance, while providing a 10 Mb/s data rate. Although the 10 Mb/s data rate is quite high considering the 66 m range, the indoor testing involved only low level artificial light interferences that can be approximated to only 200–800 lux. A high performance automotive VLC system, compatible to the IEEE 802.15.7 standard [5] is presented in [51]. This prototype is able to provide up to 200 kb/s data rates and reliable communication distances of 50 m at 99.9% confidence level, while having latencies lower than 10 ms. Although the system performances are quite impressive in terms of confidence level for 50 m range, again, the system is tested only in indoor conditions, with limited noise directly incident on the VLC receiver. The authors of [52] present the first existing V2V VLC system capable of simultaneous inter-vehicle range determination and communication. Regarding the communication part, the system is able to provide a 30 m range, at a 500 kb/s data rate and at a BER lower than 10^−6^. Again, although the results are quite impressive, the system is tested only in limited optical interferences conditions using standard fluorescent tubes driven at 50 Hz which provide a constant average illuminance of 150 lux. One of the most reliable automotive VLC systems is presented in [14]. The functionality of this system is demonstrated experimentally in vehicle-to-vehicle real-world driving scenarios. The experimental results confirmed that VLC is able to provide a reliable communication distance of up to 45 m in highway driving scenarios.

One can see that neither of the upper-mentioned works presents a VLC system capable to work in direct sun exposure. High performances results in such conditions are only reported in [16]. Nevertheless, in [16], the communication distance is only of 0.19 m. Consequently, one can see that the approach proposed in this article can significantly improve the performances of vehicular VLC systems.

## 3. Considerations Regarding the Usage of Logarithmic Transimpedance Circuits in Automotive Visible Light Communications Sensors

**Figure 1:**
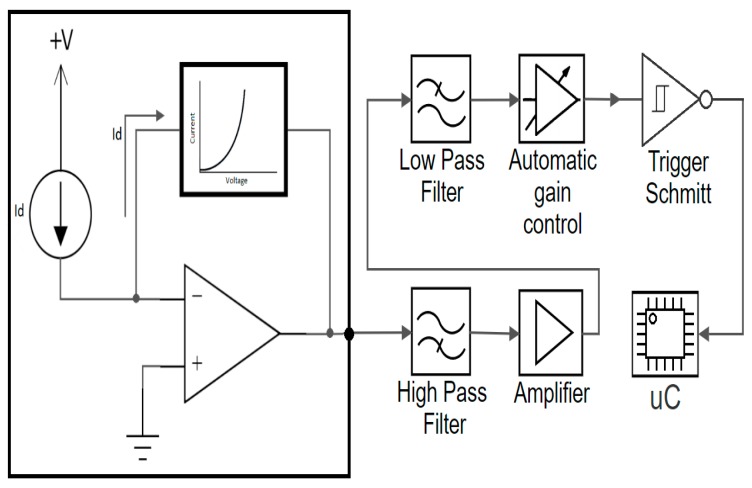
Schematic design of the VLC sensor, emphasizing the logarithmic transimpedance behavior.

Generally, a VLC sensor is based on a PIN photodiode working in the photoconductive mode and connected in a transimpedance configuration. The photodiode generates an electrical current proportional to the incident light. The transimpedance circuit transforms the produced photocurrent into a proportional voltage that is further processed in order to extract the data. Nevertheless, this article proposes a novel approach based on a transimpedance circuit having a logarithmic behavior (Figure 1). The development of the logarithmic transimpedance circuit basically follows the design of a linear transimpedance circuit except that the linear resistor (i.e., linear behavior) that establishes the gain of the circuit is replaced by a block/component/circuit that has a logarithmic behavior.

It is well known that in VLC the background light represents the most important disturbing element. The light is fluctuating in time and in space, whereas its irradiance can range from few μW/cm^2^ to more than 100.000 μW/cm^2^. Thus, the difference between the minimum value and the maximum value can expand on five decades. Consequently, selecting the working range of the transimpedance circuit can be difficult. Insufficient sensibility can lead to circuit blockage and reduced communication ranges, whereas too much sensitivity can lead to photosensitive element saturation [2]. In this case, variations of the power of the incident light will have no effect on the output voltage, which means that the incoming data will be lost. On the other hand, the output voltage for a logarithmic transimpedance circuit is given by Equation (1). We must specify here that the logarithmic circuit can introduce significant signal distortion when this solution is used in analogical transmissions. In such a case, although the dynamic range is significantly improved, the shape of the signal is affected. Then again, in digital transmissions, this signal distortion is consists of an unequal amplitude distortion, which does not have an effect on the shape of the signal.

(1) $$U_{log}=k\cdot \log\left(I_{d}\right),$$

where *U_log_* is the output voltage, *k* is a constant of proportionality, and *I_d_* is the current generated by the photodiode which is given by Equation (2); the *k* constant is introduced in the equation as the response of the circuit is not perfectly logarithmic but it has a logarithmic trend.

(2) $$I_{d}=s\cdot P_{incident\;light},$$

where *s* is spectral sensitivity of the photodiode in A/W and *P_incident light_* is the total power of the incident light.

Regarding its benefits, the logarithmic transimpedance circuit provides the VLC sensor with an expanded dynamic range and improved resilience to photoelement saturation. Thus, the sensitivity of the circuit varies logarithmically in accordance with the incident light. In dark conditions, the sensitivity is high, whereas as the power of the background light is increasing, the sensitivity is decreasing. This way, in strong light conditions, the possibility of photodiode saturation is mitigated. In addition to photoelement saturation, the incident light also generates a proportional shot noise component. Together with the internal thermal noise, these two causes represent the main sources of noise perturbing VLC. Both the thermal and the shot noise are additive white Gaussian noise sources. Additional details regarding their effects on optical communications can be found in [2,53,54].

In conclusion, the usage of a logarithmic transimpedance circuit should prevent photodiode saturation and from this point, the VLC receiver data decoding capability will be determined by the ability of the signal processing plan to extract the data.

## 4. Evaluation of the Logarithmic Transimpedance Circuit

### 4.1. Analitical Evaluation of the Logarithmic Transimpedance Circuit and Its Comparison to Linear Circuit

Before proceeding to the experimental evaluation, the next section performs an analytical evaluation of the logarithmic transimpedance circuit in order to demonstrate the concept benefits. To provide a convincing analysis, the behavior of the logarithmic solution is compared to the behavior of the classical linear solution.

**Figure 2:**
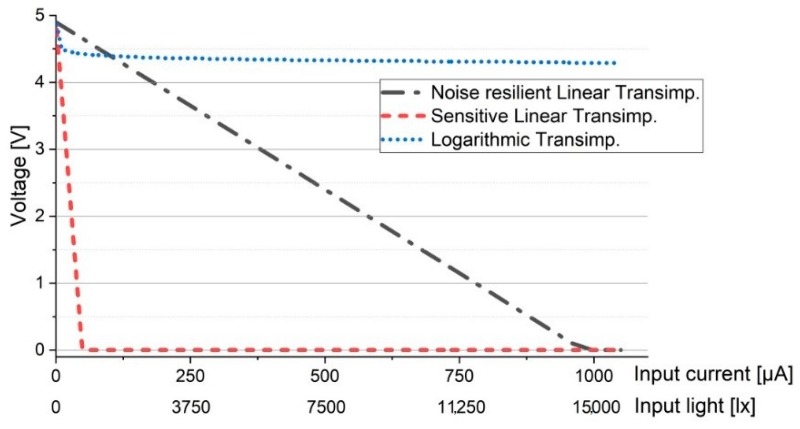
Evaluation of the logarithmic transimpedance circuit and its comparison to the noise resilient linear circuit and to high sensibility linear circuit. The simulation results show that a high sensitive linear transimpedance circuit is rapidly saturating whereas the logarithmic and the noise resilient linear prevent the photodiode saturation.

**Figure 3:**
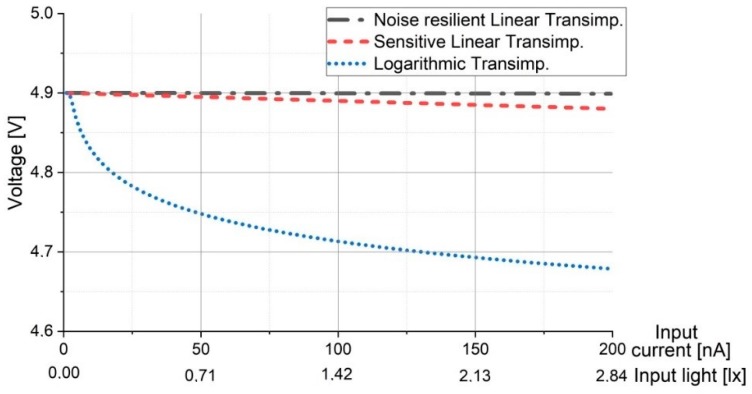
Evaluation of the logarithmic transimpedance circuit and its comparison to the noise resilient linear circuit and to high sensibility linear circuit in low lighting conditions. The simulation results show that in low lighting conditions, the noise resilient linear circuit does not respond to light intensity variations due to its low sensitivity, whereas the logarithmic transimpedance circuit automatically increases its amplification.

Thus, this section simulates the logarithmic transimpedance circuit response in varying lighting conditions, ranging from 0 lux to approx. 15.000 lux. The simulation parameters are resumed in Table 2. The 15.000 lux limit has been considered based on the impact that an IR-reject optical filter and an adaptive receiver FOV can have on decreasing the illuminance of the direct sun exposure (which may reach up to 100.000 lux) at the VLC sensor level [36,37]. In order to be even more convincing, the response of the logarithmic transimpedance circuit is compared to the response of a linear transimpedance circuit. Furthermore to provide an adequate and a fair comparison, two linear transimpedance circuits have been simulated: a linear transimpedance circuit designed in order to be highly resilient to noise (i.e., ≈ 15.000 lux) and a linear transimpedance circuit designed for high sensibility. In the first case, the simulation results (Figure 2) showed that although the resilience to noise is quite high, the sensitivity of the circuit is significantly affected, meaning that such a sensor will not be able to detect low light intensity variations, which in turn significantly affect communication distance. Now if it is to compare the sensitivity of the circuit with a VLC emitter irradiance distribution with respect to the distance, one can see that the communication distance becomes limited to just few meters. In the second case, where sensitivity was the priority, the simulation results have showed that although the higher sensitivity enables the detection of low power signals and thus, longer communication ranges, the circuit is easily saturated and consequently it is incompatible to VLC applications that imply direct sun exposure (Figure 3.).

On the other hand, in strong lighting conditions (up to 15.000 lux) the logarithmic transimpedance circuit is far from being saturated, preventing the communication blockage (Figure 2). Moreover, even in the presence of strong light, the circuit still responds to low light intensity changes, meaning that the data has the potential to be extracted (Figure 2). Nevertheless, we must admit that in such conditions, the voltage amplitude generated by the low light intensity changes (i.e., by the data signal) is very low (i.e., as low as few mV), and thus the SNR may be significantly affected. Yet, the important thing is that the circuit is not saturated, and so if proper signal processing is implemented, the data can be extracted. Another aspect which must be pointed out is that the overall amplitude response of the logarithmic transimpedance circuit in the 10–15.000 lux interval is lower than the amplitude response of the linear circuit (see Figure 2), and so an AGC circuit must be introduced to compensate the highly variable output voltage. In low lighting conditions (i.e., few lux and lower), the sensitivity and the amplitude response of the logarithmic transimpedance circuit increases, and thus its output voltage is quite similar to the one of a sensitive linear circuit.

Therefore, considering an environment where the light oscillates on 5 decades, the logarithmic transimpedance circuit provides a fair response, it has a built it flexibility, providing the sensitivity of a highly sensitive linear circuit and the robustness of a noise resilient circuit.

### 4.2. Experimental Evaluation in Strong Lighting Conditions: Confirming the Logarithmic Transimpedance Circuit Ability to Prevent the Saturation of the Photosensitive Element

**Figure 4:**
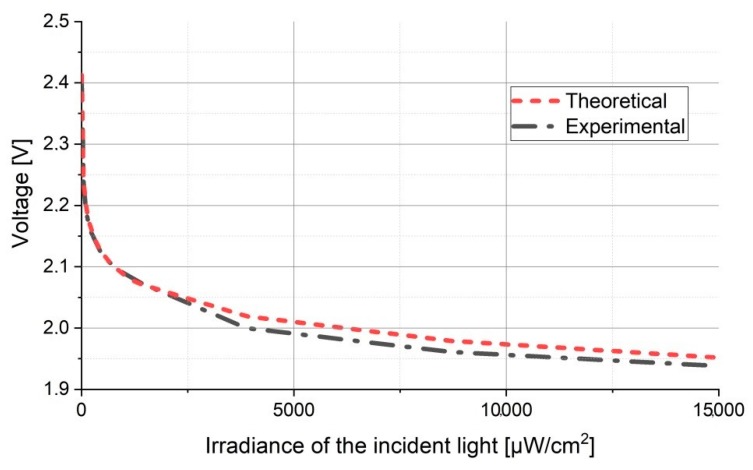
Experimental evaluation of the logarithmic transimpedance circuit ability to mitigate photoelement saturation. The graphic illustrates the circuit’s response to an increasing incident light. One can see that although the incident irradiance reached 15,000 μW/cm^2^, the circuit still responds to changes in light intensity.

Before proceeding, the first set of experiments is meant to analyze the behavior of the logarithmic transimpedance circuit and to confirm its ability to prevent photodiode saturation. For this test, the circuit is orientated toward a light source with no data transmission capability and its output voltage is analyzed as the incident irradiance is gradually increased. Table 3, Table 4 and Table 5 show the parameters of the photosensitive element, of the LED source and the measurement equipment used during the testing procedure. Figure 4 presents the results of these tests and it confirms the logarithmic transimpedance circuit’s ability to mitigate the photoelement saturation. In this case, one of the curves represents the experimental data and the other curve represents the theoretical data for a perfectly logarithmic response of the system. This plot is obtained in accordance with Equations (1) and (2).

One can see that even at 15,000 μW/cm^2^ the circuit is not saturated and that it is still able to respond to light variations. Considering the differences between the experimental results and the theoretical ones, they are due to the circuit nonlinearities. Nevertheless, these slight variations do not affect the form of the digital signal. Another observation regarding this experiment is related to the irradiance of the incident light. It is true that when the sun is in direct line of sight with the VLC receiver, the irradiance of the incident light can be even greater than 15,000 μW/cm^2^. Nevertheless, with the proper optical filters, this irradiance can be reduced to values similar to the ones that were considered in this test. Furthermore, the cost of such filters would not influence significantly the overall cost of the system. Consequently, this test confirms that the logarithmic transimpedance based VLC sensor is not saturated by background light whose power is very close to the optical power that could be incident on a VLC sensor which is directly facing the sun.

### 4.3. Experimental Evaluation of the Logarithmic Transimpedance Circuit: the Comparison with the Linear Circuit

This section presents an experimental evaluation of the logarithmic transimpedance circuit providing the comparison to a classical linear transimpedance circuit. For these tests, a VLC emitter based on a standard LED traffic light is placed 18 m away from the two transimpedance solutions and their output signals are analyzed. The parameters of the LED traffic light based VLC emitter are presented in Table 6, whereas the measurement equipment used during the testing procedure remains the one from Table 5.

**Table 6:** Summary of the LED traffic light parameters.

Parameter	Feature/Value
VLC emitter	Standard compliant LED-based traffic light: ELBA 3S1TL
Light source	5 x High power red LEDs
Total LED power	9W
Traffic light irradiance measured at 1 m	190 μW/cm^2^
LED central wavelength	642 nm (see Figure 5a)
LED spectral width	40 nm
Traffic light half angle	15˚
Traffic light diameter	200 mm
Traffic light height	200 cm

**Figure 5:**
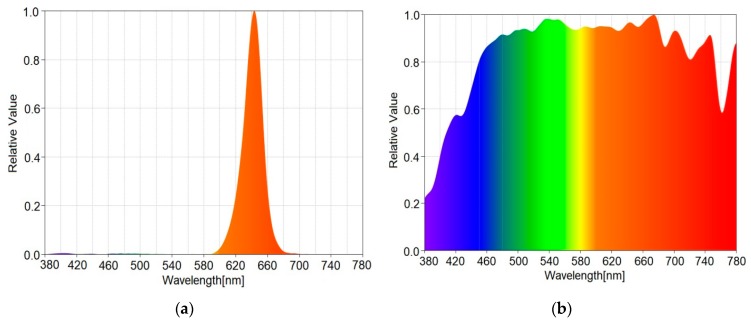
(**a**) LED traffic light spectral analysis; (**b**) Sunlight spectral analysis during the tests.

**Figure 6:**
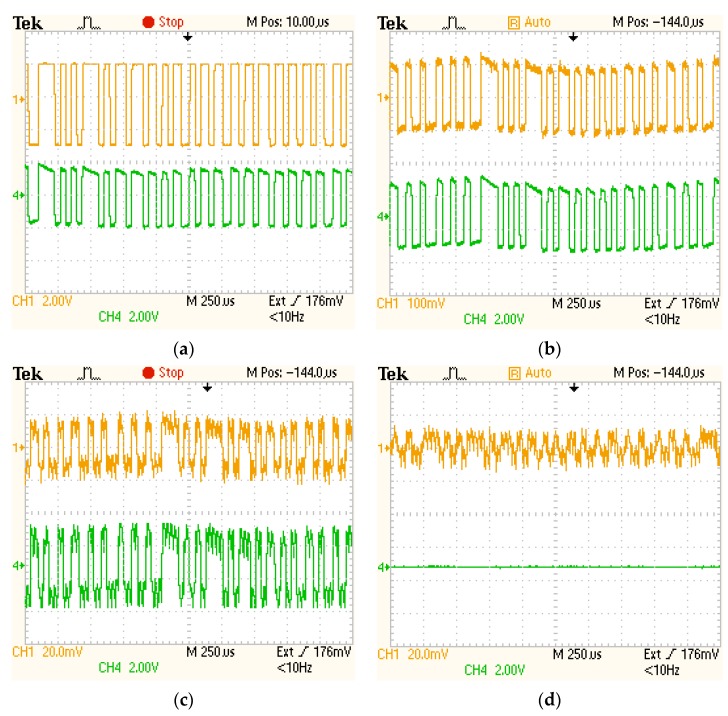
Comparative experimental evaluation showing the response of the logarithmic (Channel 1—orange) and of the linear (Channel 4—green) transimpedance circuits: (**a**). in dark conditions (no additional background light); (**b**). in high SNR conditions (incident noise irradiance of 200 μW/cm^2^); (**c**). in medium SNR conditions (incident noise irradiance of 1100 μW/cm^2^); and (**d**). in low SNR conditions (incident noise irradiance of 4500 μW/cm^2^). One can see that as the irradiance of the parasitic light is increasing, the logarithmic transimpedance circuit reduces its sensibility in order to prevent the photoelement saturation. On the other hand, the linear transimpedance circuit is saturated when the irradiance of the incident parasitic light increases above a certain limit. These experimental results confirm the benefits of the logarithmic transimpedance circuit.

At a distance of 18 m, the two VLC transimpedance circuits receive a signal having an irradiance of approximately 0.56 μW/cm^2^. In the absence of any additional parasitic light, the amplitude of the logarithmic transimpedance output signal is higher than the one of the linear transimpedance solution (see Figure 6a). The second tests have been performed in diffuse parasitic natural lighting conditions (i.e., natural light having an irradiance of about 200 μW/cm^2^). In these circumstances, the logarithmic transimpedance decreases its sensitivity and thus, the amplitude of its output signal decreased 25 times, whereas the signal provided by the linear circuit remains at the same amplitude (see Figure 6b). Next, in order to further evaluate the robustness to noise of the two solutions, a perturbing LED noise source is orientated toward the two circuits. In these conditions, the logarithmic transimpedance circuit further reduces its sensibility, and so, the amplitude of its output signal is reduced at only 40 mV, whereas the amplitude of the classical circuit is still at 3.8V (Figure 6c). At this point, the noise effects are visible on the two signals. One can see that the LED light source is generating in addition to a strong DC component, a rather strong high frequency shot noise component affecting the SNR of both circuits. So, even if the amplitude of the logarithmic transimpedance circuit is significantly lower, the SNR of the two signals is very similar. This confirms that in strong parasitic lighting conditions only the amplitude of the logarithmic transimpedance circuit is affected, whereas the SNR is similar to the one of the linear transimpedance solution. On the other hand, although the amplitude of the logarithmic transimpedance circuit output signal is significantly lower than the one of the linear transimpedance circuit, this fact does not affect the sensor’ overall performances as this effect can be compensated with the help of the automatic gain control (AGC) block. Next, the irradiance of the incident parasitic light is further increased to about 4500 μW/cm^2^. From this point, the linear transimpedance circuit is saturated and it is no longer able to receive any data. On the other hand, the logarithmic transimpedance circuit further reduces its sensibility preventing saturation. In this case, the SNR is strongly affected by the additional shot noise introduced by the parasitic light. Nevertheless, with the help of a proper signal reconditioning algorithm, the data can still be extracted.

Based on this experimental evaluation, one can see that the logarithmic transimpedance circuit provides the VLC receiver with improved resilience to noise, making this solution suitable for applications in which the VLC sensor should work in strong parasitic light exposure. As a minor drawback, this solution imposes the usage of an AGC block that compensates the variations of the data signal amplitude. Nevertheless, the AGC block is an essential component in most automotive VLC sensors, because such applications impose that the sensor is able to process signals having a very broad range of powers (i.e., the emitter receiver distance is constantly changing, and thus the power of the received data signal).

### 4.4. Experimental Evaluation in Long Range Outdoor Conditions

**Figure 7:**
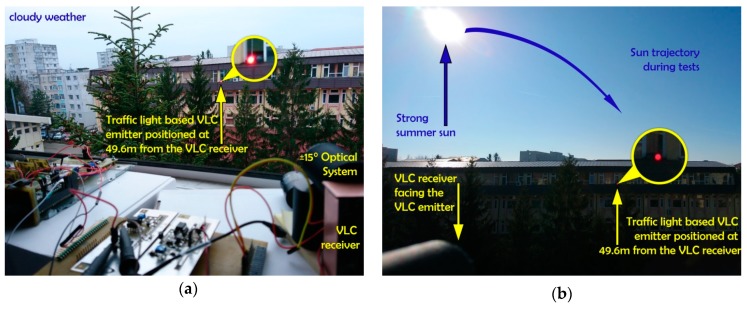

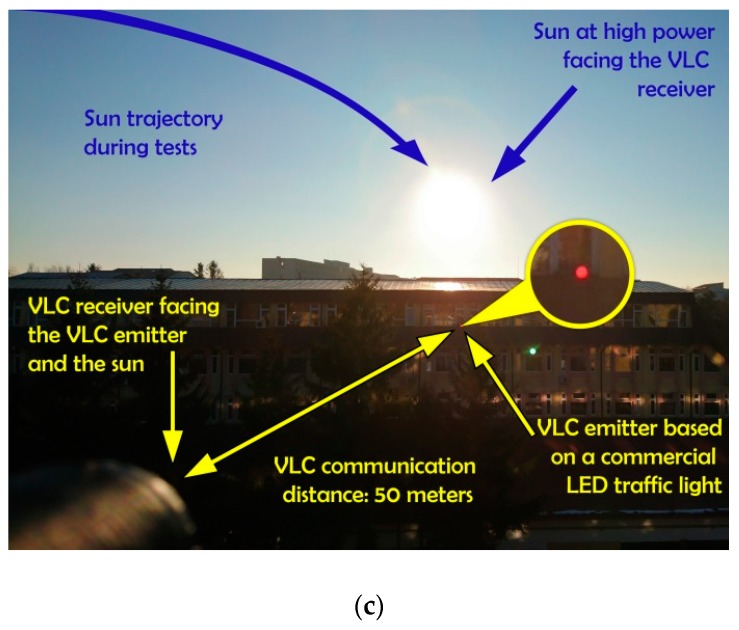
Picture showing the experimental setup for the 50 m VLC tests: (**a**) outdoor tests performed in moderate background lighting conditions; (**b**) strong sunlight conditions with the sun positioned left of the VLC receiver; (**c**) VLC evaluation in strong sunlight conditions: The VLC receiver is facing the LED traffic light VLC emitter. The positions of the VLC emitter and of the VLC receiver are static whereas the sun position is changing. During the afternoon, the sun is directly facing the VLC receiver.

The purpose of the next set of experiments is to demonstrate that the proposed logarithmic transimpedance solution is compatible to long range automotive applications and with outdoor conditions. The spectral analysis of the optical interferences during the tests is showed in Figure 5b. For these tests, the standard compliant LED traffic light is used as a VLC emitter (see Table 6). The VLC emitter is configured to broadcast data modulated using standard On-Off-Keying (OOK) modulation and Manchester coding [5]. Concerning the modulations frequencies, these tests are performed at 11 kHz. Nevertheless, modulation frequencies up to 100 kHz can be used as well. Yet, as these tests are meant to evaluate the resilience to noise, the evaluation for higher frequencies has not been addressed. In order to perform such tests, the signal provided by the logarithmic transimpedance light sensing solution is further processed using standard signal processing techniques (see Figure 1). Thus, the signal is filtered, amplified and finally converted to digital data. Then, the digital signal is introduced into a 180 MHz microcontroller and the BER is determined (in real time) by comparing the received bits with the original message. As the aim of this article is to present the benefits of the logarithmic transimpedance solution, full details regarding the signal processing are not within purposes of this article. Nevertheless, these signal processing techniques follow the general structure of a VLC sensor. These tests are performed in outdoor conditions for a communication distance of about 50 m in variate lighting conditions (see Figure 7). At this distance, the irradiance of the received VLC signal further decreases, whereas the influence of the background light becomes more stringent (i.e., lower SNR). In this case, we approximated the irradiance of the received data signal to be around 0.076 µW/cm^2^ (i.e., the approximation is made based on the optical irradiance measured at 1 m). For these tests, the VLC receiver’s Field of View (FOV) is set at 30° (i.e., ±15°). For such distances, the selected FOV is compatible to mobile applications as the solid angle between a traffic light and an on-vehicle VLC receiver is smaller than 3.5°.

The configuration used during the outdoor testing can be summarized as follows:

- LED-based traffic light VLC emitter (see parameters in Table 6);
- PIN photodiode logarithmic transimpedance circuit based VLC emitter (see architecture in Figure 1 and parameters in Table 3);
- Receiver FOV: 7,5°/15°/30°;
- Off the shelf IR-reject optical filter;
- Modulation type: OOK;
- Coding type: Manchester;
- Optical clock: 11 kHz;
- Asynchronous transmission;
- Emitter – Receiver distance: 50 m;
- Conditions: outdoor uncontrolled conditions; tests performed in different moments of the day in order to evaluate the impact of the sun for various optical power levels and relative orientations with respect to the VLC emitter;
- Measured parameters: real time BER determination;

The experimental setup for these tests is shown in Figure 7a, whereas the summary of the experimental evaluation is provided in Table 7. The results confirm that the usage of the logarithmic circuit enables the VLC sensor to provide reliable communications in outdoor conditions, achieving BER lower than 10^−6^.

**Figure 8:**
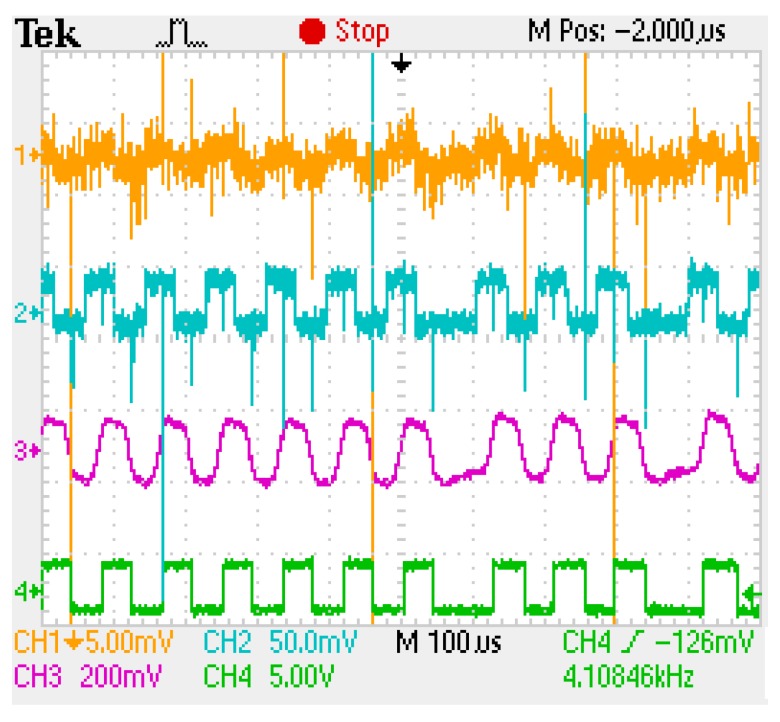
Scope screen illustrating the signal reconstruction process at the level of the different blocks of the VLC sensor: Channel 1 (orange) shows the output of the logarithmic transimpedance circuit; Channel 2 (blue) shows the output of the transimpedance circuit after a 24× amplification; Channel 3 (purple) shows the output of the filtering block; Channel 4 (green) shows the reconstructed signal which will be used in the data decoding process.

**Figure 9:**
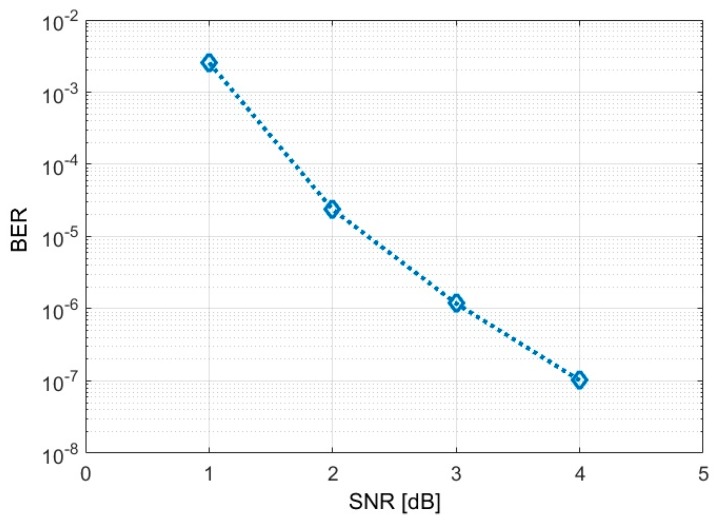
BER as a function of the SNR.

For subsequent tests, the logarithmic transimpedance based VLC receiver is evaluated in outdoor sunny conditions. For these tests, the VLC receiver is orientated toward the traffic light VLC emitter and the communication parameters are evaluated while the sun is traveling the sky. As these experiments are performed during a different moment of the day, from morning to sun fall, the VLC receiver is exposed to different sunlight powers and different incidence angles. Thus, during these testes, the VLC receiver is exposed to bright sunlight having the irradiance up to 67,250 μW/cm^2^ (Figure 7b), whereas as the sun is close to sunset, it is directly facing the VLC sensor. Consequently, one can see that these situations are very representative for the worst case VLC scenario as they imply long range communications (i.e., 50 m), outdoor conditions, strong light, and direct sun exposure. Accordingly, in order to maintain connectivity in such harsh conditions, the FOV of the receiver has been narrowed to 15° (±7.5°), whereas as the sun went toward the sunset and it was directly facing the VLC receiver, the FOV has been further narrowed to 7.5° (±3.75°). Figure 7c clearly illustrates the experimental setup showing the VLC receiver simultaneously facing the VLC emitter and the sun, whereas Figure 8 illustrates the shape of the received signal and its reconstruction process at the VLC receiver level. One can see that in strong sun exposure, the logarithmic transimpedance circuit prevents the saturation of the PIN photodiode and it is able to maintain the communication link. Nevertheless, in these conditions the logarithmic transimpedance circuit provides a signal whose amplitude does not exceeds 2–3 mV and which is very affected by noise. However, due to an adequate signal processing plan, the VLC receiver is able to reconstruct the signal and to accurately decode the data. The summary of the experimental evaluation in bright sun conditions is also presented in Table 7, whereas the BER as a function of the estimated SNR is illustrated in Figure 9. A discussion concerning the results is provided in Section 5.

## 5. Concluding Remarks Regarding the Experimental Evaluation of the VLC Sensor Based on Logarithmic Transimpedance Circuit and on Its Compatibility to Vehicular Communications

### Debate on the Proposed Vehicular VLC System Performances

The experimental results indicated that the system is able to support communication distances of 50 m while providing a BER as low as 10^−6^. Furthermore, the experiments confirmed the advantages of VLC sensors based on logarithmic transimpedance circuits, showing that this approach enables the sensors to work in very strong background lighting conditions. Consequently, the performances of such systems are more than decent compared to the ones achieved by some of the best existing VLC systems. Experimental results also reconfirmed the important influence of the receiver FOV on the communication performances. Thus, when a wide FOV is selected, the amount of background light that is captured influences the SNR, and in turn, the BER performances.

The most important contribution of this article is represented by the integration and evaluation of the logarithmic transimpedance circuit as an alternative to the linear transimpedance circuit in the automotive VLC sensors development. According to the results of the experimental investigations, the logarithmic transimpedance circuit provides the VLC sensor with improved performances in strong background light conditions as it prevents the saturation of the photosensitive element. Thus, VLC systems designed using this approach become more efficient in environments where the background light power varies significantly (i.e., automotive applications).

As demonstrated in all simulations and experiments presented in Section 4, the logarithmic transimpedance circuit is able to prevent photodiode saturation. Nevertheless, as the intensity of the incident noise increases, the amplitude of the output data signal decreases. Consequently, in strong parasitic lighting conditions associated with low power data signals, the circuit provides a low SNR data signal. From this point on, the information decoding ability is given by the capacity of the signal processing plan to eliminate the noise and to reconstruct the data signal based on a low SNR signal. For comparison, a linear transimpedance circuit operating in similar conditions would have provided no information at all, as it would have been saturated.

To further address this issue and to further improve the performances of such VLC sensors, new signal processing techniques are being investigated. Previous research has indicated that the performances of a VLC sensor can be significantly enhanced by means of digital signal processing [21]. Furthermore, the overall performances of a VLC system can be improved by developing hardware systems that are able to evaluate the environment conditions and the existing context, and which adapt their behavior (i.e., hardware design, signal processing plan, communication parameters, etc.) in accordance with the existing conditions/environment/setup [22]. Based on these facts, our current work involves the usage of combined analog and digital signal processing techniques having adaptive features controlled by a data processing unit. Thus, we are optimistic concerning the usage of VLC technology in automotive applications, not only in short range communications, in indirect light exposure, or in perfect weather, but also in unfriendly conditions. Thus, this article represents a major step forward in this direction as it is experimentally confirms a VLC link in long range, direct sunlight conditions, and with a standard power VLC emitter. The proposed VLC system based on logarithmic transimpedance has been evaluated under variable scenarios and noise conditions in order to demonstrate the compatibility of VLC systems with vehicular communication applications. The experimental evaluation of the proposed approach confirms suitability of the design and the potential of VLC technology in automotive applications.

We conclude this section by specifying that the proposed VLC system was developed mostly focusing on improving the hardware aspects in VLC design. Nevertheless, its performance can be further improved with the integration of an optical system. For example, the optical system integrated in the VLC design presented in [14] is able to provide a gain of about 15 dB. Moreover, the usage of low cost optical filters is able to reject up to 30% of the total optical noise, whereas the usage of narrow band optical filters further improves the SNR. Regarding the BER performances, the proposed system is able to achieve a row BER of 10^−6^ simply by integrating a proper light sensing solution and a decent signal processing plan. However, wireless communication systems generally use additional error correction algorithms. In VLC, Reed Solomon and Convolutional codes are generally used to improve the BER performances [5]. Thus, BERs lower than 10^−7^ – 10^−9^ can be expected. These low BER performances could be a major advantage recommending the usage of VLC technology in automotive applications.

## 6. Conclusions

In order to address the problem of long range outdoor VLC, a new VLC receiver design is herein proposed. Thus, unlike in other works, the VLC sensor is based on a logarithmic transimpedance circuit instead of a linear transimpedance circuit.

The experimental evaluation demonstrated that this solution provides the VLC receiver with enhanced resilience to noise, an extended dynamic range, and it significantly reduces the possibility of photoelement saturation. Therefore, in order to prevent the photodiode saturation, the logarithmic transimpedance circuit reduces its sensibility as the power of the incident light increases. So, from this point on, the communication performances are determined by the ability of the signal processing plan to properly reconstruct the data signal.

The experimental evaluation in long range outdoor conditions also demonstrated that the logarithmic transimpedance solution is able to provide robust 50 m communications in sunny conditions while using a standard power VLC emitter. Thus, this article provides a new argument in support of the usage of VLC technology in automotive applications.

## Figures and Tables

**Table 1:** Summary of the perturbation sources and their effect on automotive VLC systems.

Perturbation Source	Effects	Solutions	Solution Complexity
***Perturbations introduced by Natural and artificial light sources***			
Incandescent lighting sources	100 Hz perturbation and its harmonics	High pass filters;	Low
Fluorescent lighting sources	100 Hz perturbation and its harmonics	High pass filters;	Low
Sunlight	Photoelement saturation	Difficult to address;	High
	Strong DC component	High pass filters; Optical filters [16,36];	Low
	Strong shot noise (high frequency) component	Lowpass/bandpass filters; Noise subtractor [39]; Optical interference suppression based on LCD filtering [40]; Complex digital signal processing;	Moderate - high
***Perturbations introduced by Whether/atmospheric phenomena***			
Rain and Fog	Reflection, refraction, absorption, scattering – all leading to Signal Attenuation	Fresnel lens and multiple photodiodes [11];	Low
		Environment-adaptive features [22];	High
Snow	Light blockage, Attenuation	Large reception area, focal lens, multiple photodiodes [11];	Low
Dust			
***Perturbations introduced by the vehicular environment***			
Mobility	Strong variations of the emitter – receiver distance, Loss of connectivity;	Field of View Adaptivity; Automatic gain control;	Low to Moderate
Unpredictability	Loss of connectivity;	Context-adaptivity [22].	High

**Table 2:** Summary of the simulation parameters.

Parameter	Value/Feature
Photodiode Type	BPX 61
Photodiode Capacity	72 pF
Photodiode Photocurrent	70 nA/lux
Photodiode Surface	7.02 mm^2^
Light Illuminance	0–15.000 lux
Photodiode Switching Time	20 ns
Operational Amplifier Supply Voltage	5 V
Bias Voltage Non-inverting Input	4.9 V
Transimpedance solutions:	Logarithmic
	High sensitive linear circuit (R_load_ = 100 kΩ) Noise resilient linear circuit (R_load_ = 4.5 kΩ)

**Table 3:** Summary of the VLC receiver parameters during the tests.

Parameter	Feature/Value
Transimpedance circuit	Logarithmic
Photodiode type	BPX 61
Photodiode Capacity	72 pF
Photodiode Photocurrent	70 nA/lux
Photodiode surface	7.02 mm^2^
Photodiode Spectral range	400–1100 nm
Photodiode switching time	20 ns
Photodiode Sensitive area	7.02 mm^2^
Photodiode Half angle	±55˚
Photodiode Spectral sensitivity	0.62 A/W
Incident irradiance	0–15.000 μW/cm^2^

**Table 4:** Summary of the light source parameters.

Parameter	Feature/Value
Light source	High power white LED
LED power	5 W
LED Correlated color temperature	5128 K

**Table 5:** Equipment used during the tests.

Equipment Type	Equipment
Spectral analyzer	Sekonic C800
Irradiance meter	Delta Ohm HD 2302.0 with LP 471 RAD Probe
Oscilloscope	Tektronix TBS 1064

**Table 7:** Summary of the experimental evaluation of the VLC system in various outdoor conditions.

Sunlight Irradiance at Sensor Level [μW/cm^2^]	VLC Receiver FOV	Average BER	Uncontrolled Outdoor Conditions
1500 –2200	30°	<10^−6^	Cloudy afternoon
4000 –4500	30°	≈10^−4^	Bright day, with no direct visibility of the sun. See Figure 7a
12,000 –17,500	7.5°	<10^−6^	The sun sets and its irradiance is gradually decreasing.
25,000 –50,000	7.5°	10^−4^–10^−6^	Bright sun conditions, with no clouds. The sun is located right above the VLC emitter, directly facing the VLC receiver. Worst case scenario for VLC. See Figure 7c
63,000 –67,250	15°	10^−3^–10^−5^	Bright sun conditions, with no clouds. The sun was initially located left of the VLC receiver; As it travels the sky, toward sunset, its influence on the VLC receiver increases, which explains the variable BER. In this setup, the sun is not directly facing the VLC receiver. The sun is on the same axis with the VLC systems, facing the receiver. See Figure 7b
